# Repetitive Saliva Swallowing Test: Norms, Clinical Relevance and the Impact of Saliva Secretion

**DOI:** 10.1007/s00455-018-9937-0

**Published:** 2018-08-21

**Authors:** Emmelie Persson, Inger Wårdh, Per Östberg

**Affiliations:** 1 Department of Paramedicine, Brommageriatriken, Stockholms Sjukhem, Stockholm, Sweden; 2 Department of Dental Medicine and Academic Centre for Geriatric Dentistry, Karolinska Institutet, Stockholm, Sweden; 3 Department of Clinical Science, Intervention and Technology, Division of Speech and Language Pathology, Karolinska Institutet, Stockholm, Sweden; 4 Functional Area Speech and Language Pathology, Karolinska University Hospital, Stockholm, Sweden

**Keywords:** Screening, Signs of aspiration, Dysphagia, Stroke, Salivation, Normative values

## Abstract

Screening tests can be performed to identify stroke patients who require further assessment of swallowing function. The Repetitive Saliva Swallowing Test (RSST) is a screening test during which the patient is asked to swallow saliva as many times as possible for 30 s, while deglutition is counted through palpation of the larynx. This study aimed to establish normative values for three age groups of non-patients (total *N* = 120) on RSST. One patient group (*N* = 40) was also recruited from a geriatric stroke unit to assess whether RSST scores predicted outcomes on the Standardised Swallowing Assessment—Svenska (SSA-S), a clinical screening tool here used as a reference test. Since the RSST involves the swallowing of saliva, this study also measured the participants’ saliva secretion in order to examine its effect on RSST performance. This study showed that RSST results vary with age (lower among older) and gender (higher for men than women), while the number of doctor-prescribed medications, objective saliva secretion and self-assessed dryness of mouth did not affect the performance significantly. In comparison to a more extensive clinical screening procedure (SSA-S), the RSST correctly predicted 93% of negative cases and 69% of positive cases. This suggests that patients who show signs of aspiration according to SSA-S have a lower probability of detection with RSST.

## Introduction

Screening for aspiration and dysphagia among stroke patients aims to detect those patients who need to be referred for further assessment of swallowing function [1]. Screening methods should be easy to perform and detect as many patients as possible in risk of aspiration and dysphagia [2]. Several screening methods for dysphagia among stroke patients are available, such as cough test [3], water swallowing test [4], and Yale Swallow Protocol [5]. An established screening protocol is The Standardised Swallowing Assessment, SSA [6–9] that consists of an observational part to assess the patients’ general condition and a swallowing screening task. In a study comparing SSA with summative clinical judgement of swallowing function, sensitivity was 0.97 and specificity 0.90, with positive and negative predictive values of 0.92 and 0.96 [9]. The protocol is considered the best non-instrumental screening tool for dysphagia after stroke in two systematic reviews [10, 11]. However, the methodological quality was questioned in another systematic review that suggested a possible risk of bias, partially due to the fact that summative clinical judgement was used as a reference test [12]. Because of its psychometric properties and feasibility, the SSA is a suitable clinical screening tool for detecting risk of dysphagia compared to other screening methods and can be administered by nurses [12]. The Swedish translation SSA-S [13] has been validated in a study with 22 stroke patients [14] which showed good accuracy in detecting dysphagia as determined by a clinical dysphagia examination in patients with stroke in the acute phase (specificity 0.93, sensitivity 0.86). There is no consensus regarding which screening method(s) should be used in stroke departments. Several are available but further evaluation is needed.

The Repetitive Saliva Swallowing Test, RSST, is a screening test introduced in Japan by Oguchi et al. [15, 16] where the patient is asked to swallow their own saliva as many times as possible in 30 s while the assessor counts the number of swallows by palpating the larynx, or by just looking at the larynx. For normative values, they tested 60 non-patients split into two age groups; (a) healthy young participants (mean age 28.9 ± 6.9 years, range 19–47 years); (b) healthy elderly participants (mean age 68.1 ± 6.8 years, range 59–82 years), and found that the older group had significantly fewer swallows (5.9 swallows/30 s) than the younger group (7.4 swallows/30 s). They proposed that fewer than three swallows/30 s should prompt further examination of swallowing function. In a second study [16], they tested 131 patients with dysphagia to evaluate the diagnostic validity compared to video-fluorography (VF), which showed a high correlation between performance on the RSST and aspiration observed on VF. A significant difference was seen between patients with RSST ≥ 3/30 s, and patients with RSST < 3 and the occurrence of aspiration confirmed by VF. The cut-off value was set at two swallows or less/30 s. The sensitivity and specificity for aspiration were 0.98 and 0.66. The authors suggested that screening should be done with RSST. At a value of two or fewer swallows/30 s further swallowing assessment should be made to determine the appropriate treatment. Oguchi et al. [16] suggested that it is a simple and patient-safe way to screen patients for dysphagia. Unlike water swallowing test and for example meal observation, RSST does not include intake per mouth which might make it a safer screening method.

The aim of this study was to further investigate RSST by including a larger number of non-patients to get a proliferation of normative values for RSST from young adults to older participants in order to analyse the potential impact of age, gender and saliva secretion. The mucins in saliva coats all surfaces in the mouth and act as a lubricant during mastication, swallowing and speaking [17]. The secretion rate is usually higher in men than in women [18] and with ageing, histological changes occur in the salivary glands. Affoo et al. [19] found a significantly lower salivary secretion rate in individuals ≥ 60 years old compared to a younger group. The glands also become more vulnerable to the effects of medication as the glandular reserve is reduced with age [20]. The relationship between saliva production and swallowing function in a healthy population has previously been studied [21] and could not show any significant effect on swallowing function but in stroke patients, orofacial functional impairments have been reported, among those a decreased salivary flow rate [22]. Since RSST is based on the patient swallowing saliva, it may be interesting to investigate a possible link between performance on RSST and saliva secretion, because the secretion of saliva changes and decreases slightly with increasing age combined with medication [23]. The experience of dry mouth, xerostomia, has been shown to be directly proportional to the total number of medication taken per day [24] especially medications with an anticholinergic effect. It is possible that performance on RSST to some extent reflects salivation rather than actual problems swallowing, which may contribute to the low specificity. A low score on RSST need not necessarily indicate increased risk of aspiration which may lead to unnecessary restrictions on the patient’s intake per mouth. For the evaluation of its clinical relevance, RSST was tested on stroke patients and compared with the swallowing screening task from SSA-S, a non-instrumental screening tool, which was used as a reference test. An impetus to this study was also the limited available international literature on the RSST [15, 16]. Therefore, it would be interesting to add new data to the test.

## Methods

### Participants

Two groups of participants were recruited in this cross-sectional observational study; a larger group of non-patients (*N* = 120) and a smaller group of patients (*N* = 40). The non-patients were recruited through convenience sampling which meant that people were recruited among hospital staff and through the author’s personal contacts. They were then divided into groups based on age: 40 younger non-patients (age 20–40), 40 middle-aged non-patients (age 41–60) and 40 older non-patients (age 61 and older). Beyond contributing to normative values, the older group also served as a control group to the patient group. Written consent to participate was used as an inclusion criterion. Diagnosed or subjective difficulty in swallowing were used as exclusion criteria as well as previous stroke diagnosis. Participants in the test group were patients recruited from a geriatric sub-acute stroke unit at the hospital Brommageriatriken, Stockholm. Time post stroke and type of stroke varied among the patient group. Functional oral intake varied from total oral diet with no restriction to nothing by mouth, although most of the participants were somewhere in between with total oral diet but with modified consistencies. Inclusion criteria in the patient group were (i) stroke diagnosis, (ii) no occurrence of dysphagia before the onset of illness (according to the patient, relatives or journal entries) and (iii) written and/or oral consent to participate in the study. Exclusion criteria for participation were aphasia with severe impairment of understanding language or impaired cognition as a result of which the patient was not expected to be able to consent to participate in the study. The characteristics of the participants are presented in Table 1.

**Table 1:** Characteristics of the non-patients (*N* = 120), split into three age groups, and patients (*N* = 40)

Group	*N*	Age: mean (SD), range	Gender, *n* (%)
Younger	40	31.4 (5.3), 22–40	Male, 18 (45)
			Female, 22 (55)
Middle-aged	40	48.9 (5.8), 41–60	Male, 11 (27.5)
			Female, 29 (72.5)
Older	40	70.1 (7.5), 61–85	Male, 19 (48)
			Female, 21 (52)
Patients	40	83.4 (8.7), 60–99	Male, 20 (50)
			Female, 20 (50)

### Material and Measures

For objective measurement of saliva secretion, standardised cotton rolls were used which were weighed on a highly sensitive scale (VWR precision balance) before and after absorbing saliva in the oral cavity [25]. The method provides a measurement in g/min where the weight in grams is equivalent to the amount of saliva in millilitres. The threshold for dry mouth was defined as < 0.05 g/min [26]. The method has been used previously in studies where participants are not expected to be able to participate in normal salivary measurement which is more demanding for the respondent [27, 28] and has been validated in a study of dry mouth among elderly residents within retirement homes [29], referenced in the Public Health Sciences Center Linköping, 2004 [30]. For subjective measurement of saliva secretion, a modified shorter version of a test instrument with the visual analogue scale (VAS) for self-assessment of dry mouth was used [31]—tested for validity and reliability [28]. The VAS instrument has high reliability and validity in measuring subjective dry mouth. The modified version consisted of three questions: 1. How dry is your mouth. 2. How dry is your throat. 3. How dry are your lips. The RSST [15, 16] was performed on all participants. The screening instrument SSA-S [13] was used as an independent measure of risk of dysphagia in the patient group.

### Procedure

Testing of non-patients took place in the participants’ local environment such as at work or at home. Testing of the patients was performed during their stay at the stroke unit at Brommageriatriken. All participants filled out a form with their age, gender and number of prescribed medicines they were taking at the time. Saliva secretion measurements were then made, as described by Gerdin et al. [28]. A pre-weighed standardised cotton roll was placed between the right cheek and the teeth at the parotid gland opening for 1 min while the participant was at rest. Thereafter the cotton roll was weighed once again. Afterwards, participants filled in the VAS of perceived dry mouth [31]. Participants in the group of patients who needed the assistance of family members, health professionals or others did so. The swallowing assessment then followed. All participants were tested with RSST. While in a sitting position, participants were asked to swallow their saliva as many times as possible in 30 s. The number of swallows were counted by the author through palpation of the larynx which gave a ratio scale measurement: a zero count is non-arbitrary because it reflects a genuine absence of swallowing events, and the ratio is meaningful because four swallows are twice as many as two, etc. The patient group was also tested with the SSA-S [13] as an independent criterion for signs of aspiration were with or without risk of dysphagia was used as an outcome measure. The patients swallowed a teaspoon (5 ml) of water three times and then drank half a glass (1 dl) of water. Any signs of dysphagia, for example cough, change of voice or breathing quality were observed after each step. If any signs occurred, the screening ended and the patient was categorised as being in risk of dysphagia and in need of further assessment. The duration of participation in the study varied depending on the participants’ general condition between 5 and 20 min. Initially a pilot study was performed where the author and an experienced SLP colleague both performed RSST on 12 subjects from the non-patient group, representing 10% of the total number of non-patients included in the study, to calculate interrater reliability, which was excellent, ICC = 0.99 (95% CI 0.96–0.99). Thereafter all the testing was performed only by the first author (EP).

### Statistical Analysis

Statistical analyses were performed using IBM SPSS Statistics version 22. The interrater reliability was calculated using intraclass correlation (ICC). Multiple regression analysis was used to see which background factors (gender, age in years, number of prescribed medicines, saliva secretion, self-assessment of xerostomia) may affect the results of RSST. A one-way analysis of covariance (ANCOVA) was done with group as independent variable and RSST as dependent variable for a comparison of RSST between all three groups: control group (non-patients of the same age), patients with risk of dysphagia according to the SSA-S and patients without risk of dysphagia according to the SSA-S. Participant age in years was included as a covariate to control for the age difference between the control group and the patient group. In order to examine the clinical relevance of the RSST, binary logistic regression was used to allow RSST to predict the outcome of the SSA-S. The RSST score was thus the continuous predictor variable, and the binary outcome on the SSA-S was the predicted (dependent) variable in this analysis.

### Ethical Considerations

The study was approved by the Regional Ethical Review Board in Stockholm, Sweden (Dnr 2015/1458–31/2) and was carried out in accordance with the Helsinki Declaration. All participants were provided both oral and written information about the study and gave their consent before enrolling.

## Results

Data collected on the four parameters of interest for all participants are displayed in Table 2. As seen in the table, the younger and the middle-aged non-patients were quite similar on all four parameters while the older non-patients and especially the patients stand out. The latter two groups had a higher amount of prescribed medicines, scored higher on the self-assessment of xerostomia and performed fewer swallows on the RSST. Norms for younger, middle-aged and older non-patients on RSST can be seen in the last column.

**Table 2:** Data collected on all four groups of participants

Group	*N*	Number of prescribed medicines	Saliva secretion (g/min)	Self-assessment of xerostomia—mean item score	RSST—number of swallows
		Mean (SD), range	Mean (SD), range	Mean (SD), range	Mean (SD), range
Younger	40	0.15 (0.43), 0–2	0.15 (0.17), 0.04–0.87	2.32 (1.58), 0–6.33	7.90 (2.78), 4–15
Middle-aged	40	0.75 (1.03), 0–5	0.15 (0.13), 0.03–0.71	2.72 (1.94), 0–6.67	7.70 (2.52), 4–15
Older	40	2.50 (2.59), 0–12	0.15 (0.11), 0.03–0.47	3.56 (1.82), 0–7.67	6.45 (2.79), 3–13
Patients	40	5.48 (2.91), 0–16	0.10 (0.06), 0.02–0.34	4.22 (2.14), 0.67–8.67	2.88 (1.74), 0–6

### Effects of Background Variables on RSST Score

A multiple regression analysis with background factors as independent variables and RSST score as dependent variable showed a weak but significant effect which explained about 15% of the variance on RSST among the non-patients (adjusted *R*^*2*^ = .15, *p* < 0.001). Gender and age were the two background factors that significantly predicted the outcome of RSST which can be seen in Table 3. The male participants scored higher on RSST (mean 7.98, SD 2.76) than the females (mean 6.93, SD 2.69). Higher age was associated with lower RSST scores. Number of prescribed medicines, saliva secretion, self-assessment of xerostomia did not have a significant effect on RSST.

**Table 3:** Multiple regression analysis

Background variables	*B*	Beta	*T*	Sig.
Gender	1.26	0.49	2.60	0.01
Age in years	− 0.12	− 0.72	− 2.96	0.00
Number of prescribed medicines	− 0.23	− 0.16	− 1.58	0.12
Saliva secretion	2.62	0.13	1.53	0.13
Self-assessment of xerostomia	0.78	0.05	0.57	0.57

Effects of background variables on RSST scores

### Comparison Between RSST Scores for Patients and Controls

**Fig. 1:**
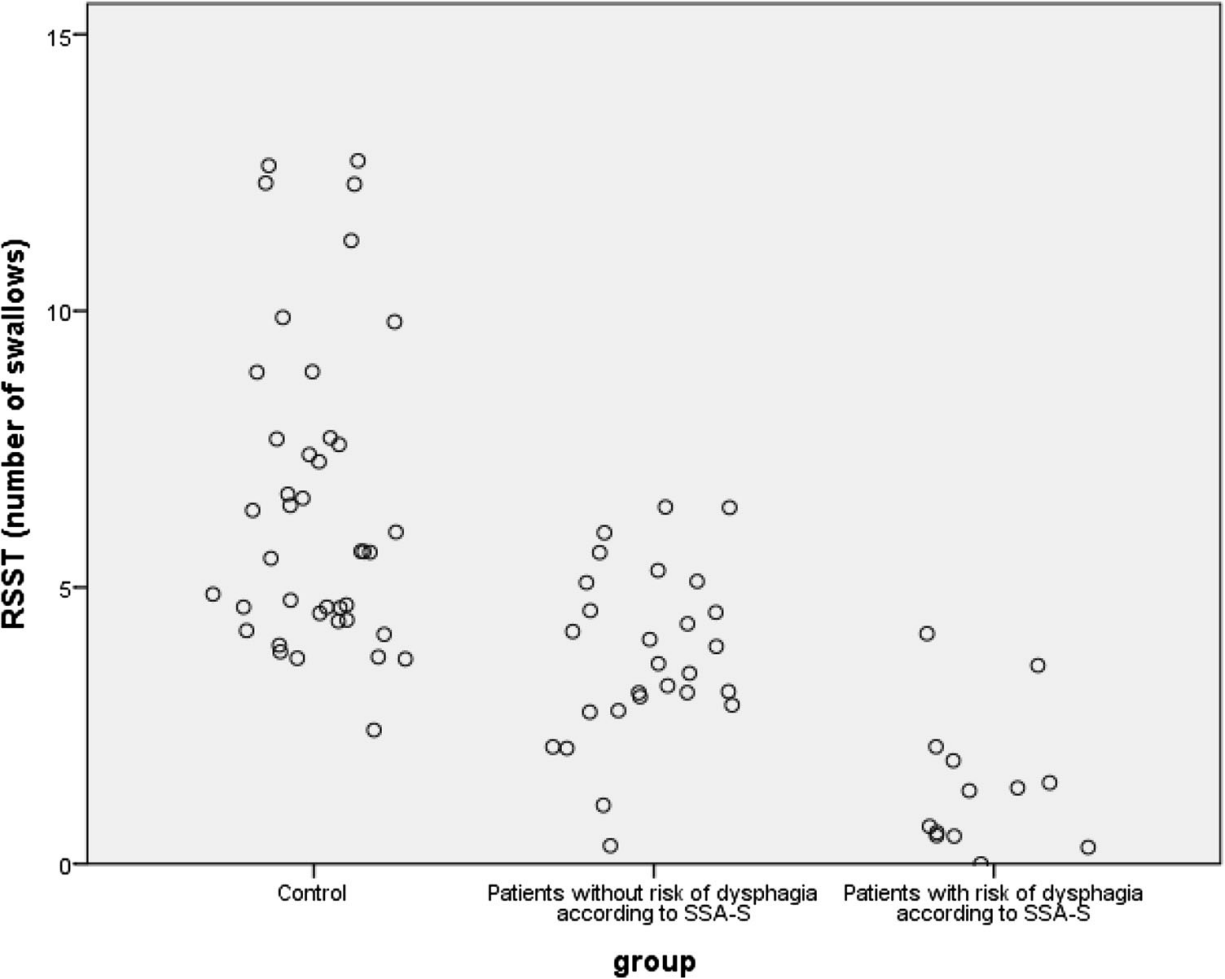
A scatter plot of the RSST scores for controls, patients without and with risk of dysphagia according to SSA-S outcome

The mean scores on RSST for all three groups: control group (non-patients of the same age), patients with risk of dysphagia according to the SSA-S and patients without risk of dysphagia according to the SSA-S can be seen in Table 4. A scatter plot of the RSST results for each group can be seen in Fig. 1. A one-way analysis of covariance (ANCOVA) with group as independent variable, RSST as dependent variable and participant age in years as a covariate showed a significant main effect of group on the RSST outcome [*F* (2,79) = 14.17, *p* < 0.001]. There was no significant effect of age as a covariate [*F* = 3.37, *p* = 0.07]. A Bonferroni post hoc test showed significant differences between all three groups. The control group thus had a significant higher RSST score than both patients with (*p* < 0.001) and without risk of dysphagia (*p* < 0.01). There was also a significant difference between patients with and without risk of dysphagia, where the patients with risk of dysphagia had fewer swallows than patients without risk of dysphagia (*p* < 0.01).

**Table 4:** RSST scores for controls, patients with and without risk of dysphagia according to SSA-S outcome

Group	*N*	Mean (SD), range
Controls	40	6.45 (2.79), 3–13
Patients without risk of dysphagia	27	3.67 (1.36), 1–6
Patients with risk of dysphagia	13	1.23 (1.24), 0–4

### Clinical Relevance of the RSST

**Fig. 2:**
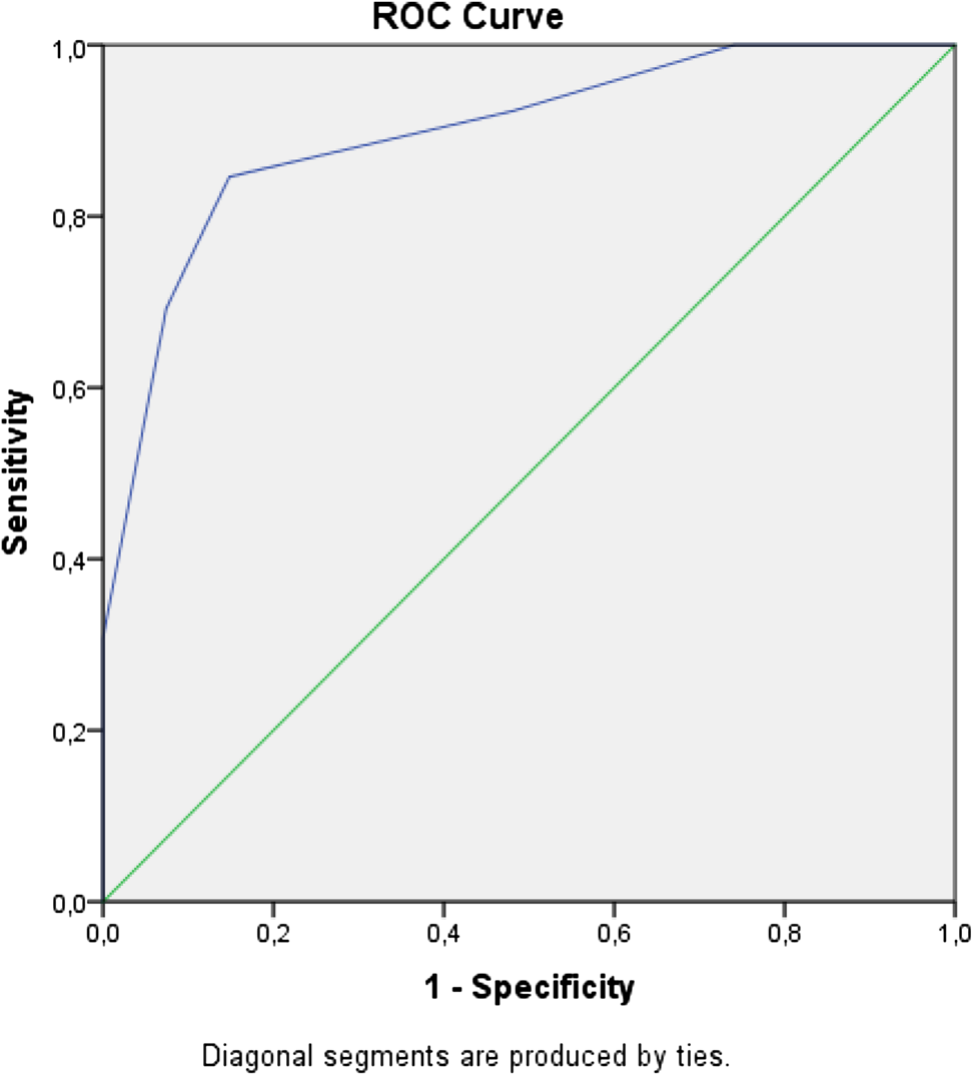
Classification of risk of dysphagia (according to SSA-S) based on RRST scores. ROC-curve with Area under the Curve (AUC) 0.897, *p* < 0.001

Binary logistic regression was used to allow RSST scores to predict the outcome of SSA-S which showed that risk of dysphagia according to SSA-S was significantly predicted by RSST (Wald *χ*^2^ = 10.98, *p* < 0.005). The overall percentage of correct predicted outcome was 85% as can be seen in Table 5. The RSST scores predicted 69% of SSA-S positive cases and 93% of SSA-S negative cases. A ROC curve, as can be seen in Fig. 2, showed good discriminative accuracy (AUC = 0.897).

**Table 5:** Classification matrix

Risk of dysphagia according to SSA-S	Risk of dysphagia according to RSST		Percentage correct
	NAD	Risk of dysphagia	
SSA-S			
NAD	25	2	93
Risk of dysphagia	4	9	69
Overall percentage			85

Prediction of risk of dysphagia (based on SSA-S) from RSST scores

*NAD* nothing abnormal detected

## Discussion

The focus of this study was to present norms for RSST from young adults to older participants and to analyse the potential impact of age, gender and saliva secretion on RSST performance. Three groups of non-patients split into different age groups showed that older participants performed a lower RSST score than younger and middle-aged participants. It was also shown that men performed better than women. Other factors such as number of prescribed medications, self-reported dryness of the mouth or measured saliva secretion did not have an effect on the RSST score. To evaluate its clinical relevance, the RSST was also tested on stroke patients and compared with SSA-S as an independent criterion. When comparing RSST to SSA-S, this study indicates that the RSST has discriminative accuracy in the diagnosis of dysphagia among stroke patients, but may not be as sensitive as the SSA-S in detecting dysphagia patients at risk of aspiration.

### Effects of Background Variables on RSST

Age and gender were the two background variables that had an impact on the number of swallows the participants made during RSST. The younger and middle-aged group was quite similar whereas the older group of non-patients performed fewer swallows on RSST. This might be expected as it has been shown in a previous study [15], perhaps due to age related bodily changes which makes it difficult to trigger as many swallows in a short time. Interestingly this cannot be explained by the tendency to have a changed saliva secretion because of age and a higher amount of medications [23]. Although the older participants tended to experience more dryness of the mouth, xerostomia, they did not have objectively decreased saliva secretion compared to middle-aged and younger participants. Studies have shown that there are weak relationships between xerostomia and hyposalivation [32]. In the present study, the collected saliva originated from the parotid gland that supplies with serous saliva that mainly flows when a stimulating agent is added. The resting and mucous saliva, that gives a lubricating feeling, mainly originates from the submandibular and minor saliva glands [33]. However, a previous study on the relationship of saliva production and swallowing function in a healthy population [21] could not show any significant age or gender differences in any of the salivary flow rates measured, even when performing two types of saliva collection procedures (submandibular and parotid saliva both unstimulated and stimulated). Neither could they show any effect on swallowing function

### Comparison Between RSST Scores for Patients and Controls

The control group performed significantly better on RSST than the patient group. Since higher age was connected to fewer swallows on the RSST and the patient group (mean age 83.4) was older than the control group (mean age 70.1) this was controlled for statistically. Age did not have a significant effect as a covariate, and there was significant differences between the patients and the control group on RSST. This means that the result was not affected by the age difference. The fact that the group of patients categorised as being without risk of dysphagia according to the SSA-S had a significantly lower RSST rate than the control group implies that the RSST is not a sensitive screening tool for impaired swallowing function. Instead it might show that the RSST perhaps also differ depending on the general state of the person being tested as we might assume that the group of non-patients acting as controls were in a generally better state than the patients. A significant difference can also be seen between the patients with and without risk of dysphagia where the patients with risk of dysphagia performed fewer swallows than the patients without risk of dysphagia. This cannot be explained by age differences but can be explained by swallowing function. The present study seems to support earlier stated limit for normal swallowing function at three or more swallows during 30 s [15] since the lowest RSST score for the non-patients was three.

### Clinical Relevance of the RSST

A screening test for risk of dysphagia should be easy to perform, have a high sensitivity for identifying as many patients as possible who are in risk of dysphagia and as high specificity as possible to rule out those without risk of dysphagia [1]. In this study, the sensitivity was 69% and specificity 93% for RSST. That means that RSST has a better chance to rule out risk of dysphagia than to detect all the patients with risk of dysphagia. A previous study [16] reported that two or less swallows during 30 s is the limit for risk of dysphagia and the need for further assessment. The present study cannot fully support that since the RSST score was between 0 and 4 (mean 1.23) among the 13 patients with risk of dysphagia according to SSA-S. Although it was only one patient who obtained RSST 3 and one that obtained RSST 4 it is still an overlap between the participants with and without risk of dysphagia which implies an uncertainty of the test’s diagnostic accuracy. In a previous study by Oguchi et al. [16] when RSST was compared to findings on video-fluorography, the results showed a sensitivity at 98% and specificity at 66% for RSST. The criterion used for dysphagia was aspiration confirmed on VF. Most of the patients who aspirated were correctly categorised by RSST, but low RSST results also occurred among some who did not aspirate. Meanwhile, when SSA-S was compared to a larger bedside battery [14], it resulted in a sensitivity at 86% and specificity at 93% for SSA-S. This shows that RSST needs to be studied further to assess its diagnostic accuracy. The result of this current study can partly be influenced by the small number of patients included, especially the small number of patients with risk of dysphagia. Only 13 out of 40 patients were considered to be in risk of dysphagia according to SSA-S. To evaluate the diagnostic validity of the RSST, one needs to use an instrumental evaluation technique such as VF or FEES as a reference test to determine to what extent the test predicts risk of aspiration and related events during swallowing. However, in this study, the SSA-S was chosen as a reference test to evaluate the clinical accuracy of the RSST because VF and/or FEES were not performed at the chosen hospital.

### Study Limitations

One limitation of the study was the use of convenience sampling for the non-patients. Convenience sampling is not optimal, but based on the conditions of the study, this was chosen for the non-patients. Swallowing ability is probably not a skill that gives an elite bias in recruitment and should therefore not be sensitive to the selection process. Another limitation as noted above, was the relatively small number of participants who were able to take part in the study and who actually were in risk of dysphagia according to SSA-S and RSST. This might have been a consequence of the narrow inclusion and exclusion criteria, which then leaves out a large number of patients in a geriatric stroke unit who are at risk of having dysphagia. It is not uncommon that patients in a geriatric stroke unit suffer from cognitive impairment, aphasia with impairment of understanding language and/or an effected general condition whereby they were excluded from the study. Those patients are at risk for dysphagia but might not be able to perform RSST because of difficulties understanding the task. Perhaps a screening procedure like SSA-S would have been more appropriate since it might be a more concrete task to swallow or try to swallow when given a teaspoon or a glass of water instead of having to swallow on a verbal cue which might be a more abstract task. The possible difficulties understanding the task might also mean that they would have failed for other reasons than impaired swallowing function if they would have been included in the study. If RSST was tested on a larger patient group and perhaps in a non-geriatric stroke unit, the specificity and sensitivity scores might have been better. The main methodological limitation of this study, however, was the use of another clinical screening procedure as a reference test rather than an instrumental assessment. The chosen reference test, SSA and the Swedish version SSA-S, both have been validated in previous studies [9, 14] by comparison to summative clinical judgement, which makes them partially validated. To validate the RSST, additional research will need to be conducted.

Further studies should include a larger number of patients and perhaps also fibre-optic endoscopic evaluation of swallowing (FEES) or VF as a comparison. One solution to the inadequacies of RSST to be tested on patients in worse general condition might be to include an observational part were the patient’s alertness and ability to sit up is noted. Both SSA-S [13] and Yale Swallow Protocol [5] include such parameters. If the patient is judged to be in a poor state, the assessment should be that the patient is not ready for feeding per mouth. It would also be interesting to perform the study on a non-geriatric stroke unit to see if there are more patients that can perform the RSST.

## Conclusion

RSST results vary with age (lower among older) and gender (higher for men than women), while the number of doctor-prescribed medications, objective saliva secretion and self-assessed dryness of mouth do not affect the performance significantly. In comparison to a more extensive screening procedure (SSA-S), RSST has good overall differential accuracy but is less sensitive to positive cases, which means that patients who show signs of dysphagia according to SSA-S might go undetected with RSST.
